# Renal Clear Cell Carcinoma Discovered Following Pyothorax: A Case Report

**DOI:** 10.7759/cureus.103633

**Published:** 2026-02-14

**Authors:** Achraf Benamou, Anouar El Moudane, Ali Barki

**Affiliations:** 1 Urology, Centre Hospitalier Universitaire Mohammed VI de Oujda, Oujda, MAR

**Keywords:** atypical presentation, clear cell carcinoma, pleural effusion, pyothorax, renal cell carcinoma

## Abstract

Renal cell carcinoma (RCC) is known for its heterogeneous clinical presentation and its potential for atypical metastatic spread. Thoracic manifestations are usually related to pulmonary or pleural metastases; however, presentation as pyothorax is exceedingly rare. We report the case of a patient in whom clear cell RCC was incidentally discovered during the etiological workup of a pyothorax. This case highlights the diagnostic challenges associated with unusual presentations of RCC and emphasizes the importance of thorough etiological investigation in pleural infections with atypical features.

## Introduction

Renal cell carcinoma (RCC) accounts for approximately 2-3% of all adult malignancies and represents the most common primary renal tumor in adults. Clear cell RCC (ccRCC) is the predominant histological subtype, accounting for nearly 70-80% of cases [1]. RCC is well known for its highly variable clinical presentation, ranging from incidentally detected small renal masses to advanced metastatic disease with systemic manifestations. Its biological behavior is characterized by a marked tendency for hematogenous dissemination and metastasis to unusual anatomical sites [2].

The lungs are the most frequent site of RCC metastasis, followed by bone, liver, brain, and adrenal glands [3]. Pleural involvement is less common and typically occurs in association with pulmonary metastases rather than as an isolated finding. Thoracic manifestations of RCC most often include pulmonary nodules, lymphangitic spread, or malignant pleural effusion. Presentation as a purulent pleural effusion or pyothorax is exceedingly rare and represents an unusual diagnostic scenario [4].

Pyothorax (pleural empyema) is most commonly caused by bacterial pneumonia, tuberculosis, thoracic surgery, or chest trauma [5]. Malignant etiologies are uncommon and may delay diagnosis when infection dominates the clinical picture. In rare circumstances, underlying malignancy may predispose to secondary infection through tumor necrosis, fistulization, or local immune dysregulation [6]. Recognizing these atypical pathways is essential, particularly when pleural infection shows an unusual course or poor response to appropriate treatment.

We report a rare case of ccRCC incidentally discovered during the etiological workup of a pyothorax, highlighting an uncommon mode of presentation and emphasizing the importance of broad diagnostic evaluation in atypical pleural infections.

## Case presentation

A 62-year-old woman with no significant past medical history was admitted with fever, dyspnea, and left-sided chest pain that had been evolving for three weeks. Clinical examination revealed decreased breath sounds and dullness to percussion over the left hemithorax. Laboratory tests showed leukocytosis and elevated inflammatory markers.

**Figure 1:**
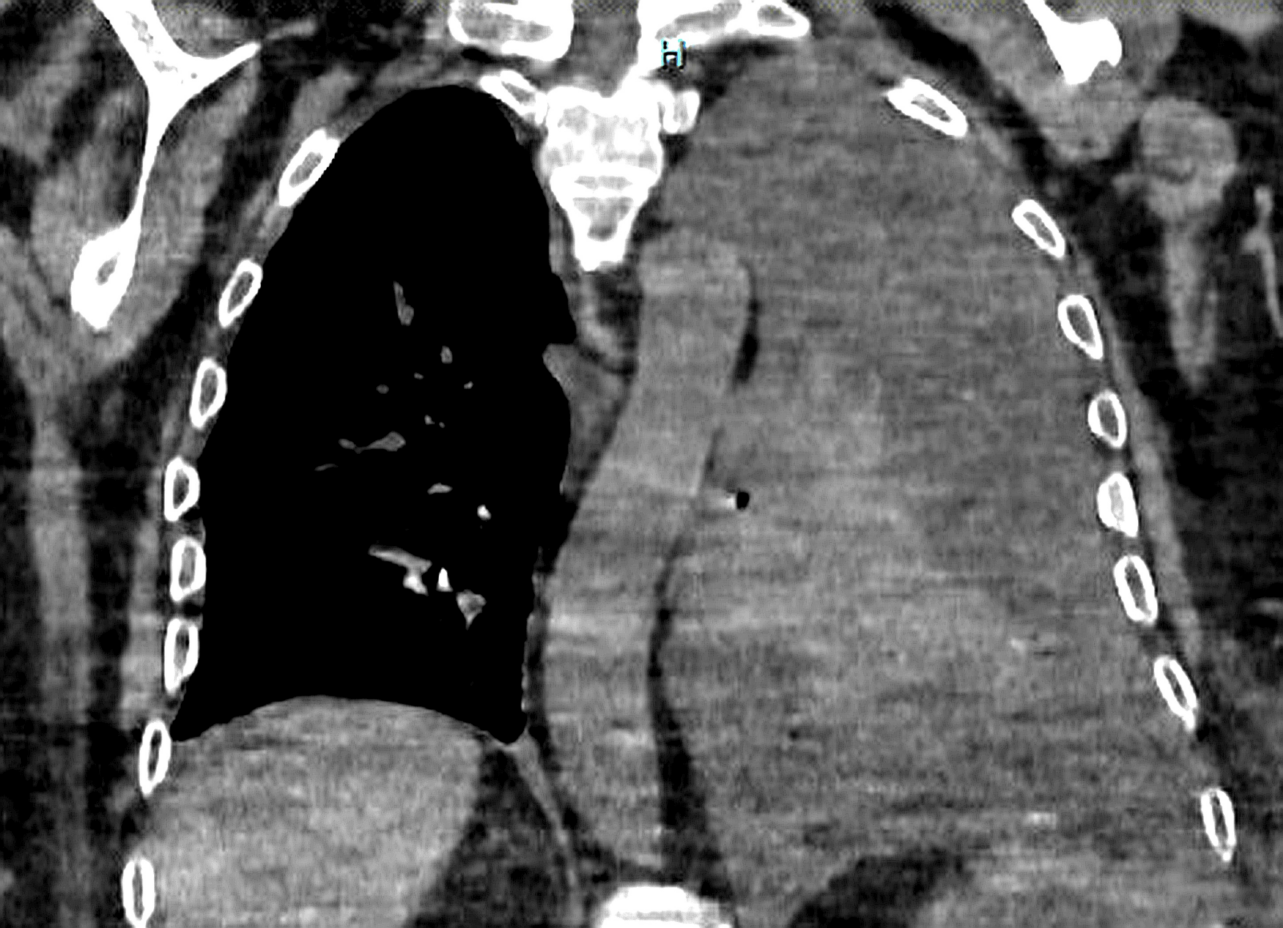
A chest CT scan revealed a large left pyothorax compressing the lung

Chest radiography demonstrated a large left pleural effusion. A thoracic CT scan confirmed a voluminous pleural effusion with imaging features suggestive of pyothorax (Figure 1). Diagnostic thoracentesis yielded purulent fluid. Microbiological cultures, including testing for* Mycobacterium tuberculosis*, were negative. The patient was hospitalized and treated with broad-spectrum antibiotics along with chest tube drainage.

**Figure 2:**
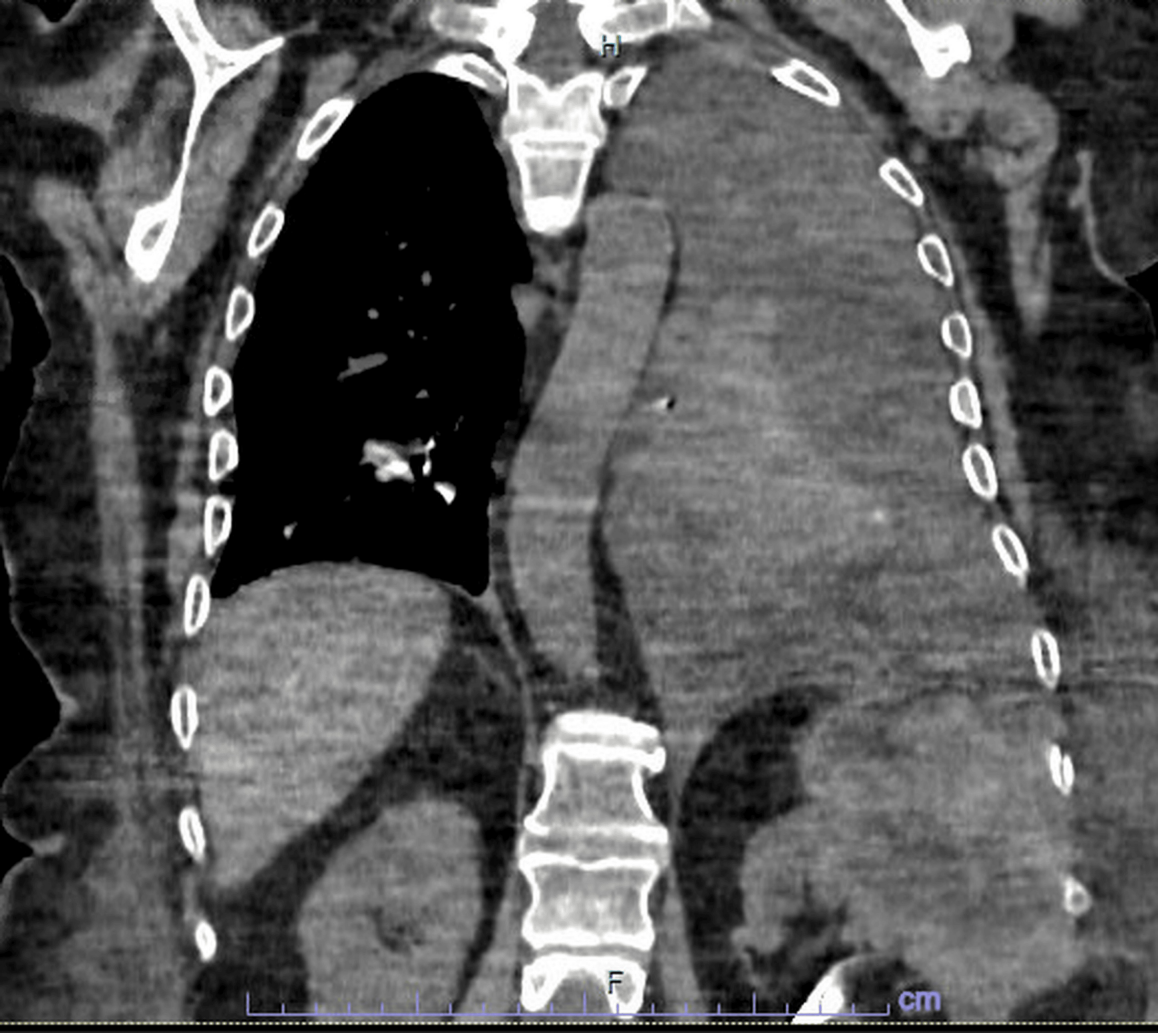
Thoraco-abdominal CT scan revealing a superinfected and fistulized left renal mass to the pleura, resulting in a large pyothorax

Because of slow clinical and biological improvement despite appropriate antibiotic therapy and effective pleural drainage, an abdominopelvic CT scan was performed as part of an extended etiological workup. This examination incidentally revealed a left renal mass with irregular contrast enhancement, central necrosis, and features suggestive of superinfection and fistulization (Figure 2).

**Figure 3:**
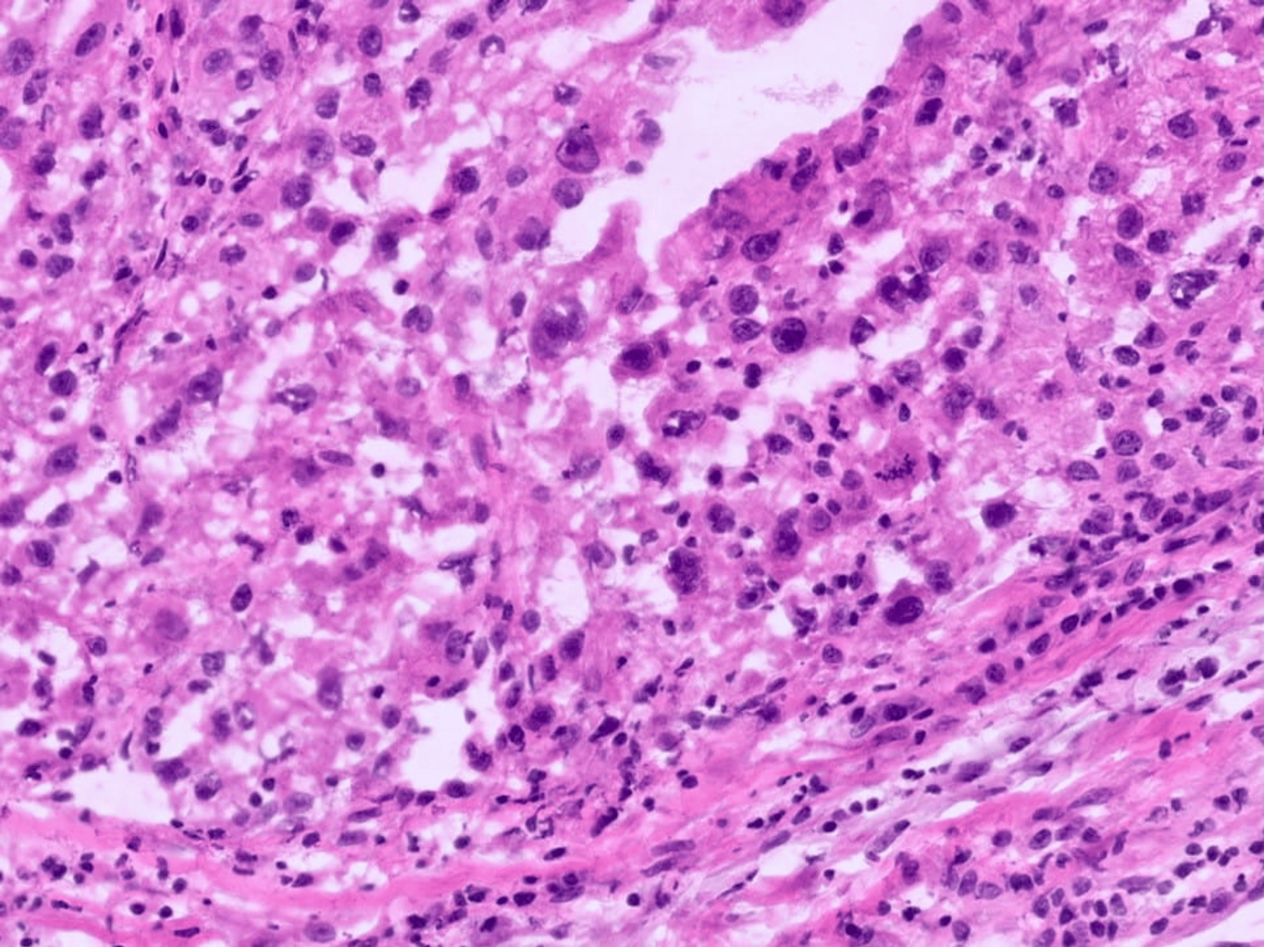
Anatomopathological image showing the appearance of a renal clear cell carcinoma with overlapping architecture and atypical cells

**Figure 4:**
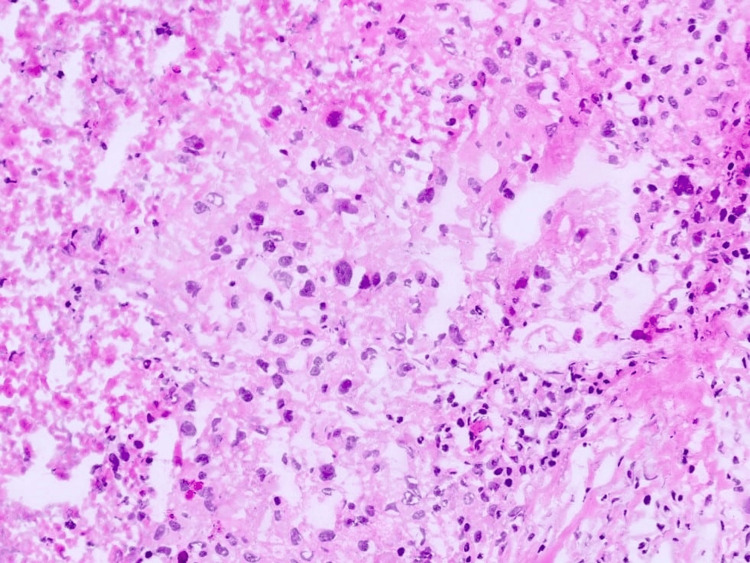
Anatomopathological image showing the appearance of a renal clear cell carcinoma with the presence of necrosis

After stabilization of the infectious process, the patient underwent radical nephrectomy. Histopathological analysis confirmed ccRCC (Figures 3, 4).

The postoperative course was uneventful, and the patient was referred to the oncology department for further management and follow-up.

## Discussion

RCC is characterized by a broad clinical spectrum, ranging from incidental detection of small renal masses to advanced metastatic disease associated with systemic symptoms. ccRCC is the most frequent histological subtype and is well known for its aggressive biological behavior and unpredictable metastatic pattern [3]. The lungs represent the most common metastatic site, followed by bone, liver, brain, and adrenal glands. Pleural involvement is relatively uncommon and typically occurs in association with pulmonary metastases rather than as an isolated manifestation [7,8].

Presentation of RCC as pyothorax is exceptionally rare and sparsely documented in the literature. Pyothorax, or pleural empyema, is classically caused by bacterial pneumonia, tuberculosis, thoracic surgery, or chest trauma. Malignancy accounts for only a minority of cases and is more frequently associated with malignant pleural effusion than with frank purulent collections [9]. In the present case, the clinical, biological, and radiological findings were strongly suggestive of a primary pleural infection, which initially diverted attention from a possible underlying neoplastic cause.

Several pathophysiological mechanisms may explain this atypical presentation. RCC is often associated with areas of tumor necrosis due to its rapid growth and abnormal vascularization. Necrotic tumor tissue may serve as a nidus for bacterial colonization through hematogenous spread, potentially leading to secondary infections at distant sites. In addition, cancer-related immune dysregulation may predispose patients to severe or unusual infections. Another possible mechanism is the presence of microscopic pleural metastases that are not detectable on imaging or cytology but may induce local inflammation and increased vascular permeability, creating a favorable environment for bacterial superinfection [10].

The diagnostic yield of pleural fluid cytology in RCC-related pleural disease is limited, with sensitivities generally lower than those observed for other solid tumors [6]. As a result, malignant causes may be overlooked when pleural infection dominates the clinical picture. Persistent or atypical pyothorax that fails to respond adequately to well-conducted antibiotic therapy and drainage should therefore prompt clinicians to broaden the diagnostic evaluation. Cross-sectional imaging of the abdomen and pelvis plays a crucial role in this context and is recommended in cases of unexplained systemic infection or when the clinical course is atypical [7,11].

From a therapeutic standpoint, management requires a multidisciplinary approach. Control of the infectious process is the first priority to reduce perioperative risk and improve general condition. Once stabilized, definitive oncological treatment can be undertaken. Surgical resection remains the cornerstone of treatment for localized RCC and provides the best chance of long-term disease control when feasible [5]. In our patient, radical nephrectomy was successfully performed after resolution of the pleural infection, allowing treatment with curative intent.

This case illustrates an atypical presentation of kidney cancer and underscores the importance of maintaining a high level of vigilance regarding the possibility of an underlying malignancy in patients with pleural infection of atypical course or undetermined etiology. Awareness of these rare presentations can help reduce diagnostic delays and improve the patient's overall prognosis.

## Conclusions

ccRCC may rarely present with atypical thoracic manifestations such as pyothorax, creating significant diagnostic challenges. When pleural infection follows an unusual clinical course or shows poor response to appropriate antimicrobial therapy and drainage, clinicians should consider the possibility of an underlying malignancy. This case underscores the importance of comprehensive etiological evaluation, including cross-sectional abdominal imaging, in unexplained or persistent pleural infections. Early recognition of these rare presentations may reduce diagnostic delay, allow timely oncological management, and ultimately improve patient outcomes.
